# Smith County First to Organize

**Published:** 1903-07

**Authors:** 


### Smith County First to Organize.

Organization of counties in line with the plan of the State and national associations is progressing well in Texas. Smith county was the first to organize. Several counties have fallen in, and Travis will be organized on the 17th, pursuant to call of Councilor Bennett. By the time the State Association meets in Austin next April it is hoped that every county will be organized and that we shall have such a meeting as never before. Whoop 'em up, boys.

